# Exercise Training Attenuates Hypertension Through TLR4/MyD88/NF-κB Signaling in the Hypothalamic Paraventricular Nucleus

**DOI:** 10.3389/fnins.2019.01138

**Published:** 2019-10-24

**Authors:** Jie Qi, Xiao-Jing Yu, Li-Yan Fu, Kai-Li Liu, Tian-Tian Gao, Jia-Wei Tu, Kai B. Kang, Xiao-Lian Shi, Hong-Bao Li, Ying Li, Yu-Ming Kang

**Affiliations:** ^1^Department of Physiology and Pathophysiology, School of Basic Medical Sciences, Xi’an Jiaotong University, Xi’an, China; ^2^Key Laboratory of Environment and Genes Related to Diseases, Ministry of Education, Xi’an Jiaotong University, Xi’an, China; ^3^School of Clinical Medicine, Xi’an Jiaotong University, Xi’an, China; ^4^Department of Ophthalmology and Visual Sciences, The University of Illinois at Chicago, Chicago, IL, United States; ^5^Department of Pharmacology, School of Basic Medical Sciences, Xi’an Jiaotong University Health Science Center, Xi’an, China

**Keywords:** hypothalamic paraventricular nucleus, exercise training, hypertension, pro-inflammatory cytokines, TLR4

## Abstract

Exercise training (ExT) is beneficial for cardiovascular health, yet the central mechanism by which aerobic ExT attenuates the hypertensive responses remains unclear. Activation of pro-inflammatory cytokines (PICs) in the hypothalamic paraventricular nucleus (PVN) is important for the sympathoexcitation and hypertensive response. We thus hypothesized that aerobic ExT can decrease the blood pressure of hypertensive rats by reducing the levels of PICs through TLR4/MyD88/NF-κB signaling within the PVN. To examine this hypothesis, two-kidney-one-clip (2K1C) renovascular hypertensive rats were assigned to two groups: sedentary or exercise training and examined for 8 weeks. At the same time, bilateral PVN infusion of vehicle or TAK242, a TLR4 inhibitor, was performed on both groups. As a result, the systolic blood pressure (SBP), renal sympathetic nerve activity (RSNA) and plasma levels of norepinephrine (NE), epinephrine (EPI) were found significantly increased in 2K1C hypertensive rats. These rats also had higher levels of Fra-like activity, NF-κB p65 activity, TLR4, MyD88, IL-1β and TNF-α in the PVN than SHAM rats. Eight weeks of ExT attenuated the RSNA and SBP, repressed the NF-κB p65 activity, and reduced the increase of plasma levels of NE, EPI, and the expression of Fra-like, TLR4, MyD88, IL-1β and TNF-α in the PVN of 2K1C rats. These findings are highly similar to the results in 2K1C rats with bilateral PVN infusions of TLR4 inhibitor (TAK242). This suggests that 8 weeks of aerobic ExT may decrease blood pressure in hypertensive rats by reducing the PICs activation through TLR4/MyD88/NF-κB signaling within the PVN, and thus delays the progression of 2K1C renovascular hypertension.

## Introduction

Hypertension is a complex disease and a risk factor for many other cardiovascular diseases. Abundant evidences now suggest that the paraventricular nucleus of hypothalamic (PVN) functions as a pivotal role in the patho- and physio-logy of hypertension (Kang et al., 2009; Wang et al., 2016; Bai et al., 2017; Li et al., 2019). Previous studies suggest that pro-inflammatory cytokines (PICs) such as TNF-α and IL-1β were increased within the PVN of spontaneous hypertensive rats (Song et al., 2014), and lead to the development of hypertension symptoms (Shi et al., 2011). PICs in the PVN is recognized as a major cause of elevating renal sympathetic nerve activity (RSNA) in hypertension.

Recently, exercise training (ExT) has been reported to be beneficial to patients suffering from hypertension when used as a supplement to pharmaceutical antihypertensive therapies (Nogueira-Ferreira et al., 2016). Research in athletes (Caselli et al., 2017), patients (Nascimento et al., 2017; Liu et al., 2018) and animals (Masson et al., 2015b; Rocha et al., 2016) also suggests that it’s beneficial in the treatment of hypertension. ExT can effectively down-regulate the PICs in the PVN, enhance the baroreflex sensitivity and lower blood pressure in hypertensive rats (Zhang et al., 2016). However, very little is known about the central mechanism through which Ext modulates PICs in the PVN and attenuates hypertension.

Recently studies have shown that TLR4 is an important receptor for the signaling transduction in the innate immune system, which affects various cardiovascular diseases such as heart failure and hypertension (Kandadi et al., 2012; Wang S.Y. et al., 2018; Dai et al., 2019; Singh et al., 2019). TLR4 has been recognized as an important regulator for NF-κB (Li et al., 2016). Growing pieces of evidence have shown that activated TLR4 within the PVN contributes to RSNA in salt-sensitive hypertension, by which the MyD88 is activated and subsequentially lead to the activation of NF-κB (Li et al., 2016; Wang M.L. et al., 2018).

Thus, to reveal the upstream signaling events that lead to decreased PICs production in the PVN following ExT, we tested the hypothesis that ExT can suppress TLR4/MyD88/NF-κB signaling in the PVN and thus attenuate two-kidney-one-clip (2K1C) renovascular hypertension.

## Materials and Methods

### Animals

All animals used in this study were approved by the Institutional Animal Care and Use Committee (IACUC) of Xi’an Jiaotong University and in accordance with the guide from National Institutes of Health for the Care and Use of Laboratory Animals (NIH Publications No. 8023, revised 1978). Adult male Sprague-Dawley rats (275–300 g) were provided by the Laboratory Animal Center of Xi’an Jiaotong University. These rats were housed with a 12 h light/dark cycle for a week of acclimatization and *ad libitum* access to standard rat chow and water. All the rats were preselected to be able to run in a small-animal treadmill before the start of the experiment (FT-200, Chengdu Techman Software Co., Ltd., Chengdu, China). After 1 week of selecting, the rats were allocated to kept sedentary (Sed) or aerobic exercise training (ExT, 50–60% of maximal exercise capacity, 5 days/week, 50 min/day) (Melo et al., 2003; Martins et al., 2005; Cai et al., 2016; Zhang et al., 2016).

### PVN Cannulae Implantation

Bilateral PVN cannulae were implanted to all rats as described in previous publications (Kang et al., 2009; Qi et al., 2016b; Huber et al., 2017). Rats were anesthetized with isoflurane. Then a stainless steel double cannula was implanted at the site of 1.2–1.6 mm caudal to the bregma, 0.5–0.7 mm lateral to the central line, and 7.0–7.4 mm below the skull surface to reach to the PVN (Liu et al., 2018).

### Experimental Design

**FIGURE 1:**
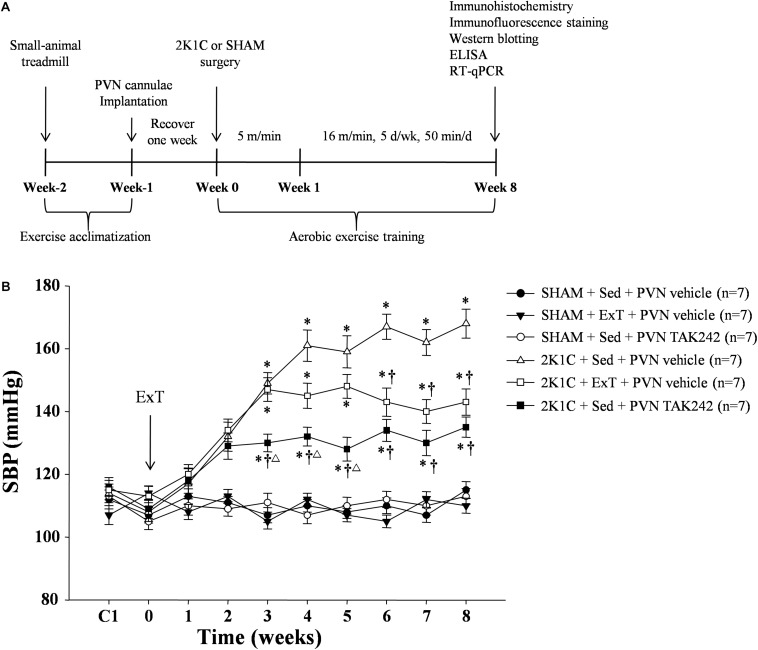
**(A)** Experimental Design. Adult male SD rats were preselected to be able to run in a small-animal treadmill before the start of the experiment. After 1 week of selecting, the rats were allocated to kept Sed or ExT. All rats were implanted with the bilateral PVN cannulae, and then were allowed to recover for a week. 2K1C or SHAM surgeries were carried out at week zero. Then the ExT groups were put on a treadmill and let run at an initial speed of 5.0 m/min, and the speed was gradually increased to 16 m/min in the second week and kept through the end of exercise. **(B)** ExT or PVN infusion of TAK242 lowered blood pressure of 2K1C hypertensive rats. ExT reduced SBP in 2K1C + ExT + vehicle in comparison with 2K1C + Sed + vehicle rats from week six till the end of the study. PVN infusion of TAK242 in the 2K1C + Sed + TAK242 rats also reduced SBP in comparing with 2K1C + Sed + vehicle group. ^∗^*P* < 0.05 versus SHAM rats (SHAM + Sed + PVN vehicle, SHAM + ExT + PVN vehicle or SHAM + Sed + PVN TAK242). ^†^*P* < 0.05 (2K1C + ExT + PVN vehicle or 2K1C + Sed + PVN TAK242) versus 2K1C + Sed + PVN vehicle. Δ*P* < 0.05 2K1C + Sed + PVN TAK242 versus 2K1C + ExT + PVN vehicle.

First, all rats were implanted with the bilateral PVN cannulae. The rats were allowed to recover for a week. Then they were randomly assigned to one of the following groups: SHAM + Sed + vehicle group, SHAM + ExT + vehicle group, SHAM + Sed + TAK242 group, 2K1C + Sed + vehicle group, 2K1C + ExT + vehicle group, and 2K1C + Sed + TAK242 group. Second, 2K1C or sham-operated (SHAM) surgeries were carried out and rats were allowed to rest and recover for three or 4 days in the first week. Then the ExT groups were put on a treadmill and let run at an initial speed of 5 m/min (20 min/d), and the speed was gradually increased to 16 m/min (50 min/d, 5 d/wk) in the second week and kept through the end of exercise (Figure 1A). After the 2K1C or SHAM surgery, an 8-week training session began: on each day, rats in each of the six groups were either left still in their cages or put on the treadmill to exercise, and then treated with bilateral PVN infusion of vehicle (artificial cerebrospinal fluid, aCSF, 400 nL) or TAK242 (TLR4 inhibitor, MCE, 240 μg in 400 nL of aCSF). The dosage is based on our previous study. During the session, blood pressure of all rats was measured weekly. After the 8-week training session, rats were sacrificed, and examinations were performed accordingly.

### Measurement of Blood Pressure

Systolic blood pressure (SBP) was measured with a non-invasive, tail-cuff system (NIBP, AD Instruments, Australia) in conscious rats as previously described (Li et al., 2016). Before the experiment started, the rats were pre-trained to accommodate the measuring procedure for at least 7 days. SBP was measured once every week till the end of the study period.

### Renal Sympathetic Nerve Recordings

Measurement of the renal sympathetic nerve activity (RSNA) parameters was performed as previously described (Kang et al., 2014; Huber et al., 2017). Briefly, rats were anesthetized with isoflurane, then the left renal nerves were isolated via retroperitoneal laparotomy and RSNA was recorded using PowerLab 4/35 (AD Instruments, Australia).

### Sample Collection

The PVN tissue was isolated following Palkovits’s microdissection procedure as previously described (Kang et al., 2011) and stored at −80°C. Blood was collected from the left carotid artery and centrifuged at 3000 rpm for 30 min. Then Plasma was removed to a clean 1.5 mL centrifuge tube and stored at −80°C for future analysis.

### Immunohistochemistry and Immunofluorescence Staining

Brains of the rats were dissected, fixed in 4% PFA at 4°C, soaked in 30% sucrose until sink, and stored at 4°C. Transverse sections with thickness of 14∼18 μm were obtained from the region approximately 1.80 mm from the bregma of the brains. Immunohistochemistry and immunofluorescence studies were performed in floating sections as described previously (Tian et al., 2019). The primary antibodies for Fra-Li (sc-253, 1:100 dilution), TLR4 (sc-293072, 1:50 dilution), MyD88 (sc-74532, 1:50 dilution), and IL-1β (sc-52012, 1:50 dilution) were purchased from Santa Cruz Biotechnology, and TNF-α (ab6671, 1:20 dilution) was purchased from Abcam. Immunohistochemistry or immunofluorescent stained sections were respectively incubated with primary antibodies at 4°C for 48 h or 24 h. For each animal, positive immunohistochemistry or immunofluorescent staining cells within the PVN were manually counted in three consecutive sections and an average value was reported. Images were taken with Nikon Eclipse 80i.

### Western Blotting

Protein was extracted from punches of the PVN as described and the western blotting analysis was performed as described (Li et al., 2014; Zhang et al., 2015). The PVN tissues were lysed in a RIPA buffer with protease inhibitor and phosphatase inhibitor cocktail. A modified BCA protein assay was used for protein quantitative. Thirty-microgram amounts of protein were separated by SDS-PAGE on 8 or 10% (wt/vol) gels and transferred on to PVDF membrane (Immobilon-P, Millipore). And then, membranes were blocked with 3% BSA in TBST buffer for 60 min at room temperature and incubated with primary antibodies at 4°C overnight. The primary antibodies for TLR4 (sc-293072, 1:100 dilution), MyD88 (sc-74532, 1:100 dilution), IL-1β (sc-52012, 1:100 dilution), and β-actin (sc-8432, 1:2000 dilution)were purchased from Santa Cruz Biotechnology, and TNF-α (ab6671, 1:100 dilution) was purchased from Abcam. The membranes were incubated with the corresponding horseradish peroxidase (HRP)-conjugated secondary antibodies (1:1000) for 60 min at room temperature. Band densities were visualized with Bio-Rad ChemiDoc XRS^+^ and analyzed using ImageJ (NIH).

### Enzyme-Linked Immunosorbent Assay (ELISA)

Plasma levels of norepinephrine (NE) and epinephrine (EPI) were measured using a rat ELISA kits (Invitrogen) following the manufacturer’s protocol (Kang et al., 2006; Qi et al., 2016a). PVN levels of TNF-α and IL-1β were measured using a rat ELISA kits (Biosource International Inc, Camarillo, California). A sandwich ELISA method was performed to measure the binding activity of free NF-κB p65 in PVN nuclear extracts using the NF-κB p65 active ELISA kit (Active Motif, United States) (Li et al., 2016).

### Quantitative Real-Time PCR (RT-qPCR)

The PVN punches were made from frozen brain sections. TLR4, MyD88, TNF-α and IL-1β mRNA expression in the PVN were examined with the following primer sequences (Table 1) and determined by RT-qPCR as previously described (Kang et al., 2006; Dange et al., 2015). RT-qPCR was performed in 96-well plates using SYBR Green qPCR Master Mix and an Mx3005P RT-qPCR Detection System (Stratagene, La Jolla, CA, United States). mRNA expression levels of the target genes were normalized to the level of GAPDH mRNA expression.

**TABLE 1:** Rat primers used for real-time PCR.

**Genes**	**Forward**	**Reverse**
TLR4	5′-GGCTGTGGAGACAAAAAT GACCTC-3′	5′-AGGCTTGGGCTTGAAT GGAGTC-3′
MyD88	5′-TCAACAAGCGAGCG CACCGT-3′	5′-TGAGCGCGACCAAC GGTAGA-3′
TNF-α	5′-ACCACGCTCTTCTGT CTACTG-3′	5′-CTTGGTGGTTTGC TACGAC-3′
IL-1β	5′-GCAATGGTCGGGAC ATAGTT-3′	5′-AGACCTGACTTGGC AGAGGA-3′
GAPDH	5′-AGACAGCCGCATCT TCTTGT-3′	5′-CTTGCCGTGGGTAG AGTCAT-3′

### Statistical Analysis

Data were represented as mean ± SEM. All data except the blood pressure were analyzed by one-way ANOVA followed by a *post hoc* Tukey test. SBP data were analyzed by repeated-measures one-way ANOVA. *P* values smaller than 0.05 was considered as statistically significant.

## Results

### ExT or PVN Infusion of TAK242 Lowered Blood Pressure

To evaluate the effect of ExT or TAK242 on blood pressure, tail blood pressure was measured weekly for all groups of rats during the entire experimental period. During the training session, the SBP among all groups of rats was similar (Figure 1B). SBP was significantly increased in the 2K1C + Sed + vehicle group rats starting at week three when compared to SHAM group rats and remained increased. ExT reduced SBP in 2K1C + ExT + vehicle in comparison with 2K1C + Sed + vehicle rats from week six till the end of the study (Figure 1B, *P* < 0.05, *n* = 7). PVN infusion of TLR4 inhibitor in the 2K1C + Sed + TAK242 group rats also reduced SBP in comparing with the 2K1C + Sed + vehicle group rats during the 3rd–8th weeks of the study (Figure 1B, *P* < 0.05, *n* = 7).

### ExT or PVN Infusion of TAK242 Attenuated RSNA

**FIGURE 2:**
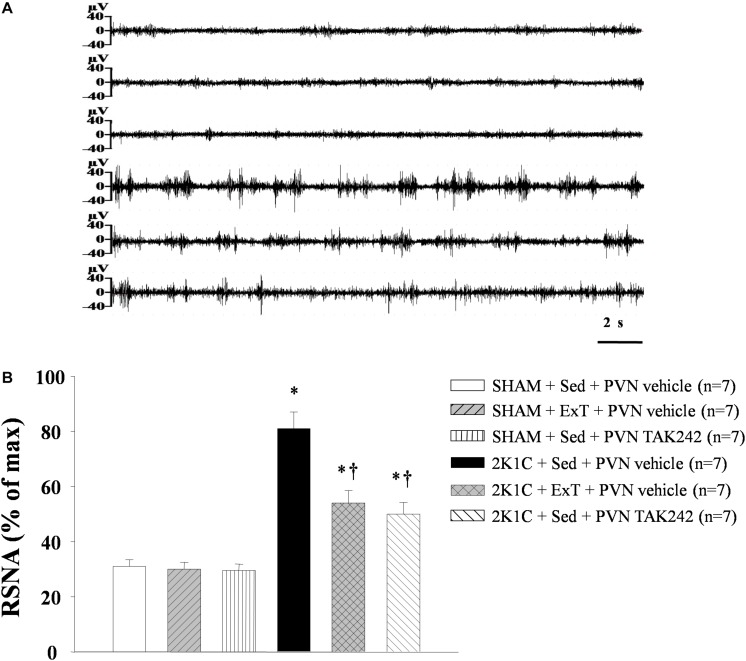
Effect of ExT or PVN infusion of TAK242 on renal sympathetic nerve activity (RSNA) in 2K1C hypertensive rats and SHAM rats. **(A)** Original tracings of RSNA. **(B)** Statistic of RSNA. 2K1C hypertensive rats had higher RSNA than in SHAM rats. ExT or PVN infusion of TAK242 attenuated RSNA of 2K1C rats. ^∗^*P* < 0.05 versus SHAM rats (SHAM + Sed + PVN vehicle, SHAM + ExT + PVN vehicle or SHAM + Sed + PVN TAK242). ^†^*P* < 0.05 (2K1C + ExT + PVN vehicle or 2K1C + Sed + PVN TAK242) versus 2K1C + Sed + PVN vehicle.

Conscious RSNA (presented as% of max) was measured 5h after rats recovered from anesthesia. 2K1C rats exhibited a higher RSNA when compared with the SHAM rats. Both exercise training or PVN infusion of TLR4 inhibitor TAK242 attenuated RSNA in 2K1C rats (Figure 2, *P* < 0.05, *n* = 7).

### ExT or PVN Infusion of TAK242 Decreased Plasma NE and EPI

**FIGURE 3:**
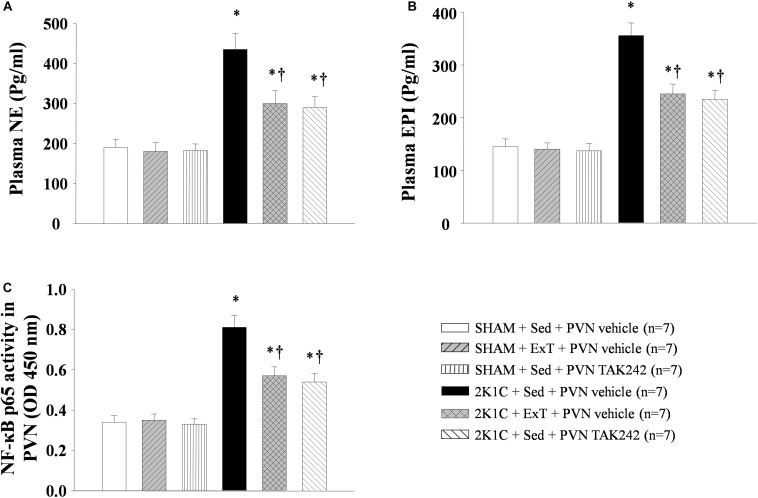
Effect of ExT or PVN infusion of TAK242 on the plasma levels of NE and EPI, and PVN level of NF-κB activation in 2K1C hypertensive rats and SHAM rats. Levels of plasma NE **(A)** and EPI **(B)**, and PVN level of NF-κB p65 **(C)** activity was shown in 2K1C hypertensive rats, sham rats and both groups after ExT or PVN infusion of TAK242. ^∗^*P* < 0.05 versus SHAM rats (SHAM + Sed + PVN vehicle, SHAM + ExT + PVN vehicle or SHAM + Sed + PVN TAK242). ^†^*P* < 0.05 (2K1C + ExT + PVN vehicle or 2K1C + Sed + PVN TAK242) versus 2K1C + Sed + PVN vehicle.

Compared with the rats in SHAM + Sed + vehicle group, 2K1C rats had higher plasma NE (a marker of sympathetic activity) and EPI levels (Figures 3A,B, *P* < 0.05, *n* = 7). After 8 weeks of exercise training or PVN infusion of the TLR4 inhibitor TAK242, plasma NE and EPI expression decreased in 2K1C hypertensive rats (Figures 3A,B, *P* < 0.05, *n* = 7). This suggests that exercise training can prevent the increase of plasma NE and EPI caused by kidney clip.

### ExT or PVN Infusion of TAK242 Reduced Neuronal Activity in the PVN

**FIGURE 4:**
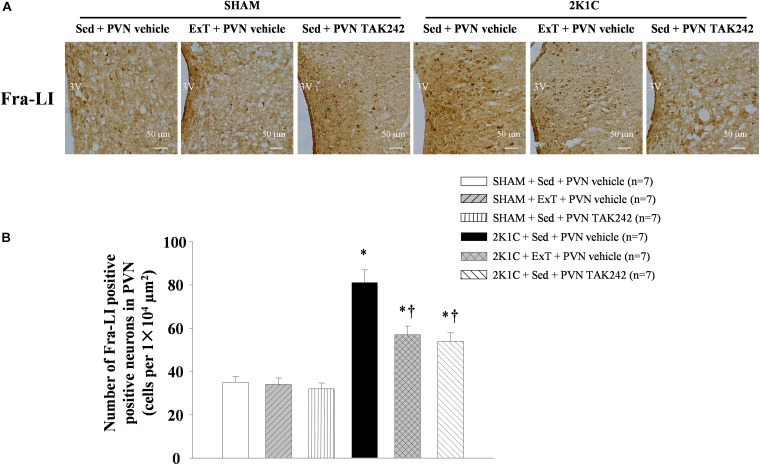
ExT or PVN infusion of TAK242 reduced PVN level of Fra-LI of 2K1C hypertensive rats. **(A)** Immunohistochemistry staining of Fra-LI in the PVN. **(B)** Statistic of Fra-LI expression in the PVN. ^∗^*P* < 0.05 versus SHAM rats (SHAM + Sed + PVN vehicle, SHAM + ExT + PVN vehicle or SHAM + Sed + PVN TAK242). ^†^*P* < 0.05 (2K1C + ExT + PVN vehicle or 2K1C + Sed + PVN TAK242) versus 2K1C + Sed + PVN vehicle.

Compared with the SHAM control group, 2K1C rats showed higher immunoreactivity of Fra-LI (an indicator of chronic neuronal activation) in the PVN (Figure 4, *P* < 0.05, *n* = 7). Exercise training of 8 weeks or PVN infusion with TAK242 reduced the number of Fra-LI^+^ cells, suggesting reduced neuronal activity in the PVN of 2K1C rats after these treatments (Figure 4, *P* < 0.05, *n* = 7).

### ExT or PVN Infusion of TAK242 Lowered NF-κB Activity in the PVN

NF-κB activity in the PVN is found to cause up-regulation of the PICs expression in hypertension. 2K1C rats showed an increase of PVN NF-κB p65 activity, while this index was significantly lowered after PVN infusion of TAK242 or exercise training for 8 weeks than that in the PVN of 2K1C + Sed + vehicle rats (Figure 3C, *P* < 0.05, *n* = 7).

### ExT or PVN Infusion of TAK242 Attenuated TLR4 and MyD88 in the PVN

**FIGURE 5:**
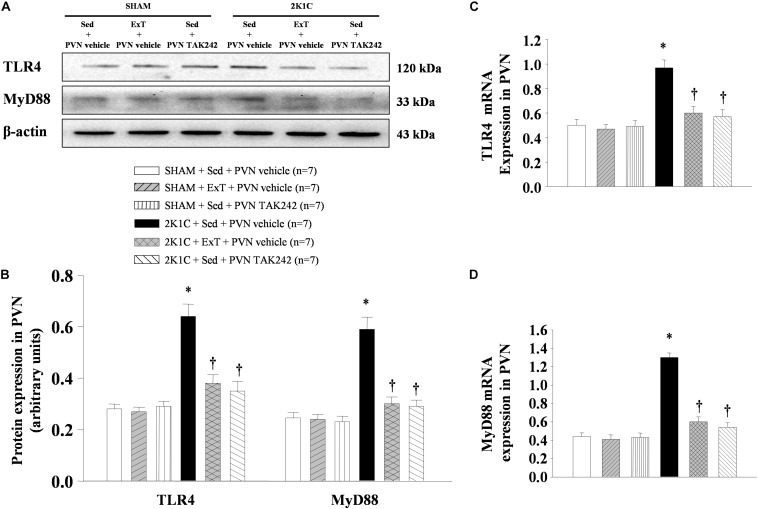
ExT or PVN infusion of TAK242 attenuated PVN levels of TLR4 and MyD88 mRNA and protein expression of 2K1C hypertensive rats. **(A)** Immunoreactive bands of TLR4, MyD88 and β-actin in the PVN. **(B)** Statistic of TLR4 and MyD88 protein expression. **(C,D)** Statistic of TLR4 and MyD88 mRNA levels. ^∗^*P* < 0.05 versus SHAM rats (SHAM + Sed + PVN vehicle, SHAM + ExT + PVN vehicle or SHAM + Sed + PVN TAK242). ^†^*P* < 0.05 (2K1C + ExT + PVN vehicle or 2K1C + Sed + PVN TAK242) versus 2K1C + Sed + PVN vehicle.

**FIGURE 6:**
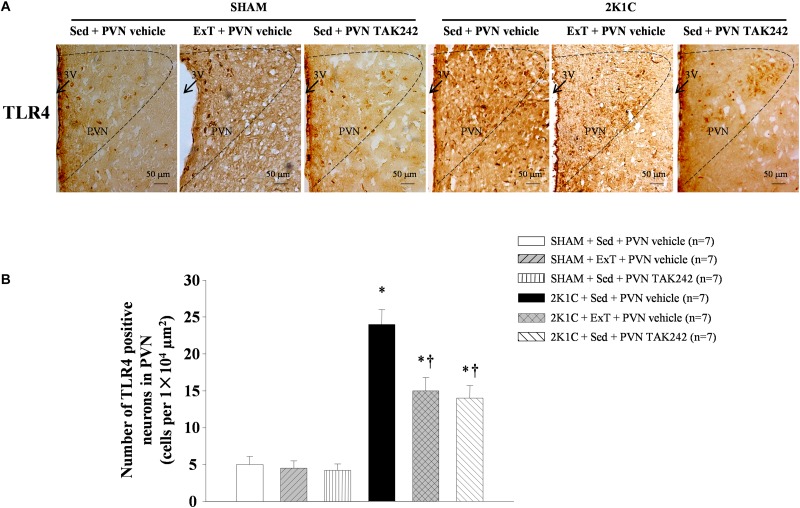
ExT or PVN infusion of TAK242 attenuated PVN level of TLR4 of 2K1C hypertensive rats. **(A)** Immunohistochemistry staining of TLR4 in the PVN. **(B)** Statistic of TLR4 expression in the PVN. ^∗^*P* < 0.05 versus SHAM rats (SHAM + Sed + PVN vehicle, SHAM + ExT + PVN vehicle or SHAM + Sed + PVN TAK242). ^†^*P* < 0.05 (2K1C + ExT + PVN vehicle or 2K1C + Sed + PVN TAK242) versus 2K1C + Sed + PVN vehicle.

Western blotting analysis and RT-qPCR of the PVN protein and mRNA showed that TLR4 and MyD88 are highly expressed in 2K1C + Sed + vehicle group rats when compared to SHAM rats (Figure 5, *P* < 0.05, *n* = 7). 8-week ExT or PVN infusion of TAK242 attenuated TLR4 and MyD88 mRNA and protein compared with 2K1C + Sed + vehicle rats (Figure 5, *P* < 0.05, *n* = 7). Immunohistochemistry stained of the PVN sections suggested that the expression of TLR4 showed a similar pattern (Figure 6). These results confirmed the efficacy of ExT or PVN infusion of TAK242 in inhibiting TLR4 and MyD88 expression within the PVN (Figures 5, 6).

### ExT or PVN Infusion of TAK242 Attenuated TNF-α and IL-1β Expression in the PVN

**FIGURE 7:**
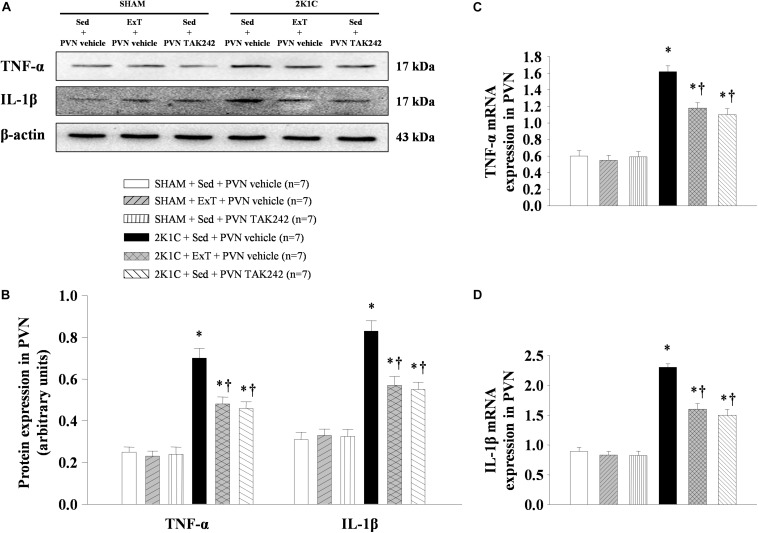
ExT or PVN infusion of TAK242 attenuated PVN levels of TNF-α and IL-1β mRNA and protein expression of 2K1C hypertensive rats. **(A)** Immunoreactive bands of TNF-α, IL-1β and β-actin in the PVN. **(B)** Statistic of TNF-α and IL-1β protein expression. **(C,D)** Statistic of TNF-α and IL-1β mRNA levels. ^∗^*P* < 0.05 versus SHAM rats (SHAM + Sed + PVN vehicle, SHAM + ExT + PVN vehicle or SHAM + Sed + PVN TAK242). ^†^*P* < 0.05 (2K1C + ExT + PVN vehicle or 2K1C + Sed + PVN TAK242) versus 2K1C + Sed + PVN vehicle.

**FIGURE 8:**
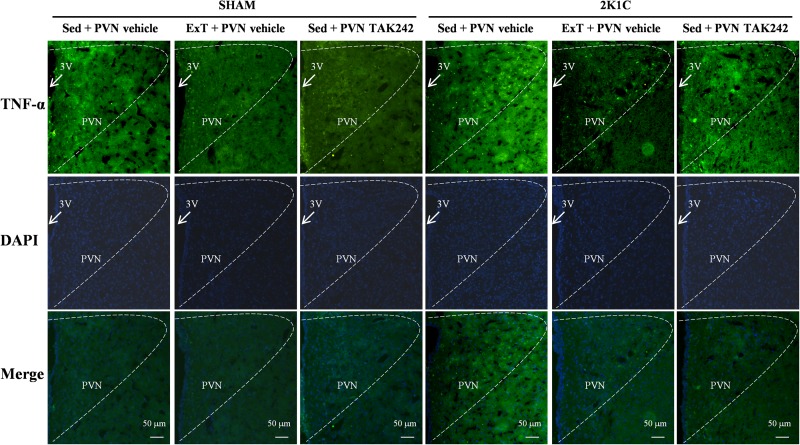
ExT or PVN infusion of TAK242 attenuated PVN level of TNF-α of 2K1C hypertensive rats. Immunofluorescence staining of TNF-α in the PVN.

**FIGURE 9:**
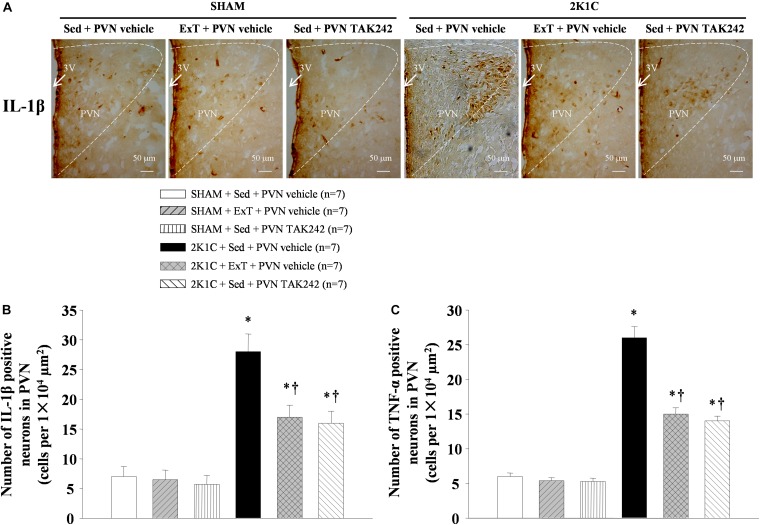
ExT or PVN infusion of TAK242 attenuated PVN level of IL-1β of 2K1C hypertensive rats. **(A)** Immunohistochemistry staining of IL-1β in the PVN. **(B)** Statistic of IL-1β expression in the PVN. **(C)** Statistic of TNF-α expression in the PVN. ^∗^*P* < 0.05 versus SHAM rats (SHAM + Sed + PVN vehicle, SHAM + ExT + PVN vehicle or SHAM + Sed + PVN TAK242). ^†^*P* < 0.05 (2K1C + ExT + PVN vehicle or 2K1C + Sed + PVN TAK242) versus 2K1C + Sed + PVN vehicle.

To test the effect of 8 weeks of aerobic ExT or PVN infusion of TLR4 inhibitor in hypertension, we respectively examined the PVN levels of TNF-α and IL-1β mRNA (Figures 7C,D) and protein (Figures 7A,B, 8, 9) by RT-qPCR, western blotting, and immunofluorescence staining. We found that 2K1C + Sed + vehicle rats had enhanced TNF-α and IL-1β mRNA and protein expression in the PVN compared with SHAM + Sed + vehicle rats (Figures 7–9, *P* < 0.05, *n* = 7). With 8-week PVN infusion of TAK242 or exercise training, these changes were attenuated (Figures 7–9, *P* < 0.05, *n* = 7).

## Discussion

The novel finding of the present study are that: (i) The TLR4/MyD88/NF-κB signaling was activated in the PVN in 2K1C renovascular hypertensive rats, and microinjection of TAK242 into the PVN inhibited TLR4/MyD88/NF-κB signaling and attenuated SBP; (ii) 8 weeks of aerobic exercise training significantly down-regulated TLR4/MyD88/NF-κB signaling transduction, down-regulated TNF-α and IL-1β protein expression in the PVN, reduced RSNA, and attenuated hypertensive response of 2K1C hypertensive rats.

Recently, it was reported that acute lipopolysaccharide (LPS)-induced TLR4 activation within the PVN contributes to excessive sympathetic activity (Masson et al., 2015a; Sun et al., 2019), and injection of TLR4 viral inhibitory peptide into the PVN suppresses the RSNA in the spontaneously hypertensive rats (Dange et al., 2015). In addition, chronic TLR4 blockade significantly attenuated blood pressure in prehypertensive rats (Caselli et al., 2017). We found that the PVN infusion of TAK-242 suppressed the TLR4/MyD88/NF-κB signaling transduction in the PVN, induced a reduction in number of Fra-LI^+^ neurons (a marker of chronically activated neurons), and attenuated SBP and RSNA in 2K1C hypertensive rats. These findings from this study show that the TLR4 activation within the PVN contributes to the 2K1C renovascular hypertensive response.

It is also well established that TLR4/MyD88 signaling pathway is directly upstream the NF-κB signaling pathways (Wang et al., 2019). Our present study showed that the TLR4 blockade causes downregulation of NF-κB activity in 2K1C hypertensive rats. NF-κB activation is the major regulator in the production of PVN TNF-α and IL-1β (Melo et al., 2003), and contributes to exaggerated sympathetic activation in high salt-induced hypertension (Caselli et al., 2017). In summary, abundant evidence shows that 2K1C renovascular hypertensive rats had significant upregulation of TLR4/MyD88/NF-κB signaling transduction within the PVN and contributes to the pathogenesis of hypertension.

Both our lab and others have reported that ExT can lead to decrease in blood pressure in high salt-induced hypertension and has been recognized as a supportive method for treatment of hypertension (Zhang et al., 2016). Our results further support these ideas and suggestions that aerobic ExT can delay the progression of hypertension. At the same time, compared with the 2K1C-Sed group, ExT group showed a significantly decrease in the expression of TLR4, MyD88, and PICs. It also led to suppressed NF-κB p65 activation in the PVN. These results are in consent with the previous findings that TLR4, MyD88, and TNF-α were upregulated within the PVN of spontaneously hypertensive rats (Li et al., 2016), and then resulted in increased sympathetic nerve activity (Dange et al., 2015).

More importantly, when we compare the rats from the 2K1C-Sed-TAK242 group and 2K1C-ExT-vehicle group, we found that reduction in the SBP of the 2K1C-ExT-vehicle group was milder than the 2K1C-Sed-TAK242 group during the 3rd–5th weeks. It reached a similar level from the 6th week. And the reduction of SBP remained similar between the two groups. These results suggest that aerobic ExT is as effective as TAK242 on preventing the alleviation of the blood pressure, though not as efficiently. There are no comparable changes in the protein levels of TLR4, MyD88, PICs, and activation of NF-κB p65 within the PVN between ExT and TLR4 inhibitor at week eight. Collectively, this study reveals that ExT could attenuate RSNA and blood pressure, and inhibit TLR4/MyD88/NF-κB signaling in the PVN of 2K1C hypertension.

**FIGURE 10:**
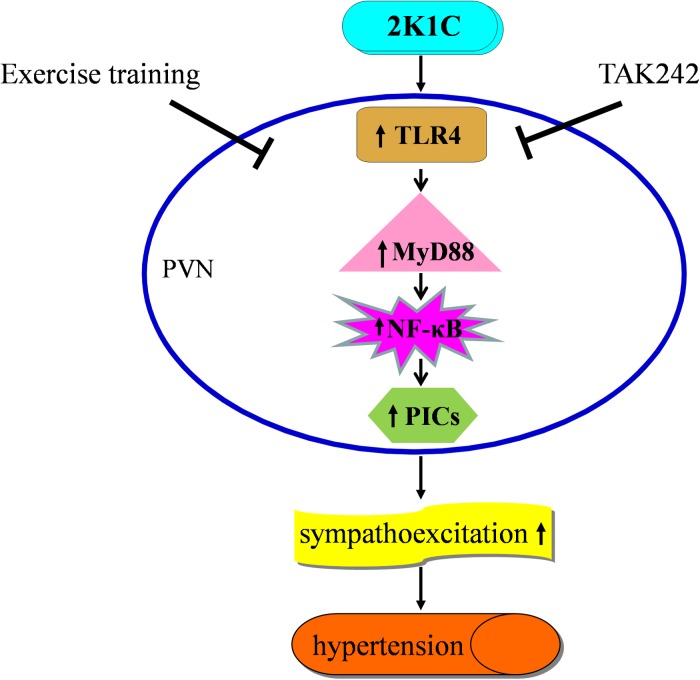
The schematic showing the mechanism by which exercise training attenuates hypertension. 2K1C induced hypertension increases TLR4/MyD88/NF-κB signaling in the PVN, which results in the overloads of PICs, causes sympathoexcitation and eventually accelerate the progression of hypertension. ExT can reverses this pathophysiological process of hypertension by attenuating TLR4/MyD88/NF-κB signaling in the PVN.

We did not investigate the effect of PVN infusion of TLR4 inhibitor within ExT group rats at present. Our present results suggest that aerobic ExT appears to have a beneficial effect on suppressing sympathetic activity and improving blood pressure. Schematic illustration of our findings (Figure 10) shows the central mechanism by which ExT may decrease 2K1C renovascular hypertension.

## Conclusion

In this study, we demonstrated that aerobic ExT is beneficial for hypertension treatment through the modulation of TLR4/MyD88/NF-κB signaling in the PVN.

## Data Availability Statement

The raw data supporting the conclusions of this manuscript will be made available by the authors, without undue reservation, to any qualified researcher.

## Ethics Statement

All animals used in this study were approved by the Institutional Animal Care and Use Committee (IACUC) of Xi’an Jiaotong University and in accordance with the guide from National Institutes of Health for the Care and Use of Laboratory Animals.

## Author Contributions

Y-MK and JQ designed the study. JQ, KK, H-BL, and YL drafted the manuscript. JQ, L-YF, X-JY, J-WT, T-TG, and K-LL performed all experiments and the data analysis. All authors reviewed the manuscript.

## Conflict of Interest Statement

The authors declare that the research was conducted in the absence of any commercial or financial relationships that could be construed as a potential conflict of interest.

## Abbreviations

2K1C, two-kidney: one-clip
aCSF: artificial cerebrospinal fluid
EPI: epinephrine
ExT: exercise training
IL-1 β: interleukin-1 beta
MyD88: myeloid differentiation factor 88
NE: norepinephrine
NF- κ B: nuclear factor-kappa B
PICs: pro-inflammatory cytokines
PVN: hypothalamic paraventricular nucleus
RSNA: renal sympathetic nerve activity
SBP: systolic blood pressure
Sed: sedentary
TLR4: toll-like receptor 4
TNF- α: tumor necrosis factor-alpha
